# Insecticide-treated durable wall lining (ITWL): future prospects for control of malaria and other vector-borne diseases

**DOI:** 10.1186/s12936-017-1867-z

**Published:** 2017-05-22

**Authors:** Louisa A. Messenger, Mark Rowland

**Affiliations:** Department of Disease Control, Faculty of Infectious Tropical Diseases, London School of Hygiene and Tropical Medicine, London, UK

**Keywords:** Insecticide-treated durable wall lining, Malaria, Leishmaniasis, Chagas disease, Vector control, Insecticide resistance

## Abstract

While long-lasting insecticidal nets (LLINs) and indoor residual spraying (IRS) are the cornerstones of malaria vector control throughout sub-Saharan Africa, there is an urgent need for the development of novel insecticide delivery mechanisms to sustain and consolidate gains in disease reduction and to transition towards malaria elimination and eradication. Insecticide-treated durable wall lining (ITWL) may represent a new paradigm for malaria control as a potential complementary or alternate longer-lasting intervention to IRS. ITWL can be attached to inner house walls, remain efficacious over multiple years and overcome some of the operational constraints of first-line control strategies, specifically nightly behavioural compliance required of LLINs and re-current costs and user fatigue associated with IRS campaigns. Initial experimental hut trials of insecticide-treated plastic sheeting reported promising results, achieving high levels of vector mortality, deterrence and blood-feeding inhibition, particularly when combined with LLINs. Two generations of commercial ITWL have been manufactured to date containing either pyrethroid or non-pyrethroid formulations. While some Phase III trials of these products have demonstrated reductions in malaria incidence, further large-scale evidence is still required before operational implementation of ITWL can be considered either in a programmatic or more targeted community context. Qualitative studies of ITWL have identified aesthetic value and observable entomological efficacy as key determinants of household acceptability. However, concerns have been raised regarding installation feasibility and anticipated cost-effectiveness. This paper critically reviews ITWL as both a putative mechanism of house improvement or more conventional intervention and discusses its future prospects as a method for controlling malaria and other vector-borne diseases.

## Background

In recent years considerable reductions in global malaria burden have been achieved by scaling-up key diagnostic, treatment and preventative measures [1]. Long-lasting insecticidal nets (LLINs) and indoor residual spraying (IRS) remain the cornerstones of malaria vector control, both targeting indoor feeding and resting mosquito vector populations [2–5]. Long-term effectiveness of these strategies is currently under threat from widespread emergence of insecticide resistance to pyrethroid LLINs [6, 7], as well as to other chemical classes used for IRS [8, 9]. Furthermore, maintaining high coverage at the community-level of either intervention can be operationally challenging. Universal coverage (UC) campaigns of LLINs have been adopted as the standard of care by most National Malaria Control Programmes (NMCPs) [1]; however, net usage is known to decline during hot seasons [10–12], and LLIN efficacy and durability under field conditions [13, 14] and rates of household attrition are also of increasing concern [15, 16]. In some epidemiological settings, IRS can be highly effective [1, 17] but the short residual activities of most insecticide formulations [18] render it logistically demanding and economically unsustainable for many endemic countries [19]. To maintain and consolidate gains and to transition towards malaria elimination and eradication [20], there is a growing impetus to develop alternate or complementary interventions [4, 5, 21], novel insecticide classes [22, 23], combinations [24, 25], formulations [26, 27] and cost-effective, scalable mechanisms of delivery [28–30], as well as to evaluate a potential role for concurrent housing improvement in disease control [31–33].

## Initial experimental development and evaluation of insecticide-treated housing materials

Insecticide treatment of house or shelter materials was first pioneered as a method to control malaria during humanitarian emergencies in countries affected by war [34–37]. Impregnation of utilitarian tents or tarpaulins with deltamethrin was intended to circumvent the logistical difficulties of achieving high coverage with IRS or insecticide-treated nets (ITNs), producing high rates of mosquito mortality in experimental platform studies and pilot malaria control projects in Pakistan [35–37]. Early experimental hut evaluations of pyrethroid (deltamethrin or permethrin) and non-pyrethroid (pirimiphos-methyl, organophosphate or bendiocarb, carbamate) treated plastic sheeting (ITPS) as an interior wall liner, indicated that this intervention functions in a similar manner to IRS against host-seeking vectors entering indoors and alighting on walls either before or after blood-feeding, or if blocked from feeding by a mosquito net (Table 1). Only limited personal protection from biting was observed when ITPS was evaluated alone, suggesting disease control would instead be achieved through a ‘mass effect’ on vector density and longevity at the community-level [38, 41, 42, 46–48]. Depending upon the excito-repellant properties of different insecticides used to treat ITPS, some studies also reported increased deterrence rates and exophily among susceptible mosquito populations, demonstrating the potential to directly interrupt human-vector contact, further contributing to a reduction in malaria transmission [38, 41, 42, 46]. For the majority of entomological parameters, ITPS efficacy was correlated with intervention surface area, with increasing coverage affording higher rates of mortality, deterrence and blood-feeding inhibition [38, 39, 46].

**Table 1:** Summary of experimental hut trials demonstrating the impact of insecticide-treated housing materials on malaria vector control

Field site, country, trial type	Intervention(s)	Insecticide (dosage)	Intervention coverage	Control(s) (dosage)	Major malaria vector species^resistance status^	Entomological parameters^b^						References
						Mortality	Deterrence	Exiting rates	Blood feeding inhibition	Personal protection	Impact on insecticide resistance	
Afghan refugee camp, Pakistan, experimental platforms	Impregnated polythene tarpaulins	Deltamethrin	Full coverage^a^	Untreated polythene tarpaulin	*An. subpictus* ^ND^, *An. stephensi* ^ND^	High mosquito mortality (86–100%); no significant differences between interventions	ND	ND	No impact on blood feeding (~20% for all interventions)	ND	ND	[36]
		(45 mg/m^2^)										
	Sprayed polythene tarpaulins	(30 mg/m^2^)										
	Impregnated polythene tents	(45 mg/m^2^)										
Afghan refugee camp, Pakistan, experimental platforms	Polyethylene canvas	Deltamethrin (ND)	Full coverage	Untreated canvas tent	*Anophelines* (spp. grouped)^ND^	Increased mosquito mortality relative to control (51 vs. 26%, respectively)	No significant reduction in mean no. of mosquitoes relative to control (7 vs. 19, respectively)	ND	Reduced blood feeding relative to control (9 vs. 46%, respectively)	ND	ND	[37]
Bobo Dioulasso, Burkina Faso, experimental West African huts [50]	Polyethylene sheeting	Permethrin (2% w/w)	Ceiling only	Untreated polyethylene sheeting	*An. gambiae* ^r^	Mortality correlated with coverage (20% for two walls; 45% for four walls; 46% for four walls + ceiling covered)	Deterrence correlated with coverage (28% for two walls; 43% for four walls; 46% for four walls + ceiling covered)	All treatments highly repellent (induced-exophily 68–78%)	No significant impact on blood feeding; level of inhibition correlated with surface area covered (10% for two walls vs. 27% for four walls + ceiling)	ND	Mortality and blood feeding inhibition *kdr* ^r^/*kdr* ^r^ < *kdr* ^r^/*kdr* ^s^ + *kdr* ^*s*^/*kdr* ^s^ (19 vs. 64% and 12 vs. 62% for four walls + ceiling, respectively)	[38]
			Two walls									
			Four walls	Untreated control								
			Four walls + ceiling									
Cotonou, Benin, experimental West African huts	Polypropylene mesh	Bendiocarb (200 mg/m^2^)	Top thirds of walls	Deltamethrin-treated mosquito net (ITN; 45 mg/m^2^)	*An. gambiae* ^r^	Mortality proportional to wall surface area covered (80% vs. 100% for upper third of wall or full coverage, respectively)	No significant reduction in mean no. of mosquitoes in full coverage hut relative to control (202 vs. 206, respectively)	ND	High levels of blood feeing inhibition; no significant increase when combining wall treatments with ITNs compared to ITNs alone (100% vs. 94%, respectively)	ND	ND	[39]
			Full coverage	Untreated mosquito net								
Bobo Dioulasso, Burkina Faso, experimental West African huts	Polypropylene sheeting (ITPS)	Bendiocarb (400 mg/m^2^)	Upper thirds of walls	Deltamethrin LLIN (PermaNet^®^ 2.0; 55 mg/m^2^)	*An. gambiae* ^r^	Significantly higher mosquito mortality when interventions used in combination (ITPS + LLIN: 73% vs. ITPS alone: 53%)	ND	Significantly higher vector exophily when interventions combined (LLIN + IRS: 61%; ITPS + LLINs: 50%)	Significant blood feeding inhibition only when ITPS combined with LLIN (58%) relative to untreated control	ND	Frequency of *ace*-*1* ^*R*^ allele significantly higher among heterozygote survivors from individual IRS and ITPS treatments but not when combined with LLIN	[41]
				IRS (bendiocarb; 400 mg/m^2^)		Mortality similar for partial coverage of ITPS vs. full coverage with IRS (53% vs. 42%, respectively)						
				Untreated mosquito net								
Bobo Dioulasso, Burkina Faso, experimental West African huts	Polyethylene sheeting (ITPS)	Permethrin (2% w/w)	Full coverage	Holed permethrin LLIN (Olyset^®^; 2% w/w)	*An. gambiae* ^r^	Significantly higher mosquito mortality when ITPS used in combination with LLIN (60%) compared to alone (34%)	No significant reductions in mean no. of mosquitoes in ITPS huts without (443) or with untreated nets (309-315), relative to control (422)	Significant increase in exophily for single (ITPS alone: 80%; LLIN alone: 77%) and combined interventions (ITPS + LLIN: 79%)	Combined use of ITPS + LLIN did not significantly increase blood feeding inhibition over LLIN alone (75% vs. 82%, respectively)	Combined use of ITPS + LLIN significantly increased personal protection over LLIN alone (88% vs. 16%, respectively	Significantly more *kdr* ^r^/*kdr* ^r^ dead with LLIN (55%) and ITPS + LLIN (67%) than ITPS alone (17%)	[42]
				Intact or holed untreated mosquito net								
				Untreated control								
Muheza, Tanzania, experimental East African huts [51]	Polyester wall hangings (NWH)	Pirimiphos methyl (1 g/m^2^)	Ceiling only	Untreated control	*An. gambiae* s.l.^s/r(c)^ *, An. funestus*	*An. gambiae* and *An. funestus* mortality significantly higher for p-methyl NWH than deltamethrin NWHs (92% vs. 11% and 78% vs. 6%, respectively for two walls)	Significant reductions in mosquito entry for p-methyl (65–95%) and deltamethrin (50–56%) treated NWH	Significantly increased exiting rates in NWH huts compared to untreated control	Limited effect on blood feeding rates (52–77%) relative to untreated control (64–67%)	ND	ND	[46]
			Two walls									
			Four walls			*An. gambiae* and *An. funestus* mortality significantly higher for two walls than ceilings only (59 and 39%, respectively)	Deterrence increased with increasing coverage (65–77% vs. 92–95% for two walls vs. four walls + ceiling)					
			Four walls + ceiling									
						No improvement in mosquito mortality when coverage increased beyond two walls						
		Deltamethrin (55 mg/m^2^)	Two walls									
Tiassalé, Côte d’Ivoire, experimental West African huts	Polyethylene wall lining (WL)	Pirimiphos methyl (1 g/m^2^)	Four walls	Holed deltamethrin LLIN (PermaNet^®^ 2.0; 55 mg/m^2^)	*An. gambiae* s.s.^r^	Significantly higher mortality with *p*-methyl WL than pyrethroid WL (66% vs. 32%, respectively)	Significant reductions in mosquito entry for p-methyl WL/NHW only when combined with LLIN (59%/65% vs. 28%/3%, respectively)	Significantly increased exiting rates for p-methyl WL (53%) and p-methyl NWH + LLIN (59%), relative to untreated control (29%)	Limited effect on blood feeding rates (82–94%) relative to untreated control (95%), unless combined with LLIN (9–13%)	Limited personal protection for p-methyl WL/NWH relative to untreated control (4%/0%), unless combined with LLIN (93%/92%)	Significantly higher numbers of *ace*-*1* ^*R*^ heterozygote (*RS*) and homozygote (*RR*) survivors compared to susceptible homozygotes (*SS*) following exposure to p-methyl WLs/NHWs	[47]
			Four walls + ceiling									
				Holed untreated mosquito net		No improvement in mosquito mortality when p-methyl WL/NWH coverage increased from walls only (66%/49%) to walls + ceilings (56%/69%)					Combined WL and LLIN did not limit the selection of *ace*-*1* ^*R*^ compared to WL alone	
	Nylon NHW	Pirimiphos methyl (1 g/m^2^)	Four walls									
			Four walls + ceiling	Untreated plastic sheeting		No increase in mosquito mortality when WL/NHW combined with LLINs (72%/61% vs. 61%/53%, respectively)						
	Polyethylene WL (ZeroVector^®^)	Deltamethrin (175 mg/m^2^)	Four walls									
Bobo Dioulasso, Burkina Faso, experimental West African huts	Polyethylene WL	Pirimiphos methyl (1 g/m^2^)	Four walls	Holed deltamethrin LLIN (PermaNet^®^ 2.0; 55 mg/m^2^)	*An. gambiae* s.s.^s/r*(c)*^	Significantly higher mortality with p-methyl WL than pyrethroid WL (>95% vs. 40%, respectively)	Largest reductions in mosquito entry for pyrethroid WL and p-methyl WL when used in combination with LLIN (74 and 62%, respectively)	Significantly increased exiting rates for p-methyl WL (53%), relative to untreated control (33%)	Blood feeding significantly reduced when p-methyl WL/NWHs combined with LLIN (91%/90% vs. 50/50%, respectively)	Personal protection for p-methyl WL/NWH relative to untreated control (56%/72%), increased when combined with LLIN (95%/94%)	Significantly higher numbers of *ace*-*1* ^*R*^ survivors (100%) following exposure to p-methyl WL alone, compared to susceptible vectors (32%)	[48]
			Four walls + ceiling									
				Holed untreated mosquito net		Significantly higher mortality with p-methyl WL/NHW either alone or in combination with LLIN (100% for all)					Combined WL and LLIN limited the selection of *ace*-*1* ^*R*^ compared to WL alone	
	Nylon NHW	Pirimiphos methyl (1 g/m^2^)	Four walls									
			Four walls + ceiling	Untreated plastic sheeting		No significant increase in mortality when pyrethroid WL combined with LLINs (48% vs. 40%, respectively)						
	Polyethylene WL (ZeroVector^®^)	Deltamethrin (175 mg/m^2^)	Four walls									
			Four walls + ceiling									

*IRS* indoor residual spraying, *ITN* insecticide-treated net, *ITPS* insecticide-treated plastic sheeting, *LLIN* long-lasting insecticidal net, *ND* not described, *NWH* net wall hangings, *r* resistant to one or more insecticides under investigation, *s* susceptible to one or more insecticides under investigation, *WL* wall lining

^a^Full coverage defined as four inner walls in experimental huts or all interior surfaces in a λ-shaped tent, as applicable

^b^Entomological parameters reported relative to untreated control, unless otherwise specified

^c^Resistant to pyrethroids but susceptible to organophosphates

## Initial community-level trials of insecticide-treated housing materials

Following preliminary trials of experimentally-treated plastic materials (Table 1), commercial ITPS (ZeroFly^®^) was originally produced by Vestergaard Frandsen (Switzerland) as high density laminated polyethylene sheets containing deltamethrin (55 mg/m^2^). Based on LLIN technology, the insecticide is incorporated into the polymer during manufacture and diffuses to the surface slowly, in a controlled fashion, acting as a long-lasting insecticide reservoir. Initial community-level evaluations of ZeroFly^®^ ITPS in temporary labour shelters and villages in India [40, 43] and among displaced populations in Sierra Leone [44] and Angola [45] supported the entomological outcomes reported by experimental hut trials, achieving significant reductions in malaria incidence (Table 2). Similar observations of the impact of coverage on intervention effectiveness were observed in Sierra Leone, where protective efficacy from malaria improved from 15 to 60% when ITPS coverage increased from ceiling only to include all four tent walls [44]. However, when carbamate-treated ITPS was evaluated in combination with UC or targeted LLIN distribution among rural houses in Benin, no additional malaria protection was reported, potentially attributable to limited wall coverage (only the upper thirds of walls were covered due to insecticide safety concerns), and the short residual activity of a single treatment of bendiocarb [21].

**Table 2:** Summary of community-level trials demonstrating the impact of insecticide-treated housing materials on malaria control

Field site, country, trial type	Intervention(s)	Insecticide (dosage)	Intervention coverage	Control(s) (dosage)	Major malaria vector species^resistance status^	Impact on vector populations^c^	Impact on disease incidence^c^	References
Orissa, India, community-level^a^	Polyethylene sheeting (ITPS)	Deltamethrin (55 mg/m^2^)	Full coverage^b^	Untreated plastic sheeting	*An. culicifacies* ^s^, *An. fluviatilis* ^s^	Significant reductions in mosquito house entry (80–89%), vector indoor population density (95–100%), blood feeding (75%) and parity rates (74–77%)	Significant reduction in malaria incidence (65–70%)	[40]
				Untreated control		Increased immediate (56%) and delayed (100%) mosquito mortality and induced exophily (41%)		
						Human blood index decreased to 0		
Uttar Pradesh, India, community-level (temporary labour shelters)	Polyethylene sheeting (ITPS)	Deltamethrin (265 mg/m^2^)	Full coverage	Untreated plastic sheeting	*An. culicifacies* ^s^, *An. fluviatilis* ^s^	Significant reductions in indoor vector population density and blood feeding, both to 0%	Significant reduction in malaria incidence to 0%	[43]
Liberian refugee camps, Sierra Leone, community-level (temporary shelters)	Polyethylene sheeting (ITPS)	Deltamethrin (55 mg/m^2^)	Ceiling + roof	Untreated plastic sheeting	*An. gambiae* s.l.^s^, *An. funestus* s.l.^s^	ND	Protective efficacy from malaria infection of 60% and 15% for full or partial ITPS coverage, respectively	[44]
			Four tent walls + ceiling				Significant increase in time to first malaria infection among full ITPS coverage group	
							Significant increases in mean Hb concentration in both intervention groups	
Ouidah-Kpomassè-Tori Bossito, Benin, community-level	Polypropylene sheeting (ITPS)	Bendiocarb (200 mg/m^2^)	Upper thirds of walls	Deltamethrin LLIN (PermaNet^®^ 2.0; 55 mg/m^2^) (targeted coverage to pregnant women and <6 years)	*An. gambiae* s.l.^s/r^, *An. funestus* s.l.^ND^	No significant reductions in human biting rate, sporozoite rate or EIR for all interventions	No significant reductions in malaria incidence, prevalence or parasite density for ITPS + LLIN, UC of LLIN or LLIN + IRS compared to targeted LLIN	[21]
						Significantly greater proportions of parous mosquitoes and indoor resting vectors in ITPS + LLIN villages		
	IRS	Bendiocarb (400 mg/m^2^)	All house walls			*kdr* allele frequency increased in all intervention groups		
	PermaNet^®^ 2.0 LLIN	Deltamethrin (55 mg/m^2^)	Universal coverage					
Balombo, Angola, community-level	Polyethylene sheeting (ITPS) (ZeroFly®)	Deltamethrin (360 mg/m^2^)	Full coverage	Deltamethrin LLIN (PermaNet^®^ 2.0; 55 mg/m^2^)	*An. funestus* ^ND^ and other minor anopheline spp.	Significant reductions in indoor vector population density (82% for ITPS + LLINs; 78% for IRS; 73% for WL) and intensity of mosquito bites in most intervention villages, measured using anti-*Anopheles* saliva IgG antibodies levels	Significant reductions in malaria incidence (58% for ITPS + LLINs; 54% for IRS; 51% for WL)	[45]
				IRS (lambdacyhalothrin; 25 mg/m^2^)				
	Polyethylene WL (ZeroVector^®^)	Deltamethrin (175 mg/m^2^)						

*EIR* entomological inoculation rate, *Hb* haemoglobin, *IRS* indoor residual spraying, *ITPS* Insecticide-treated plastic sheeting, *LLIN* long-lasting insecticidal net, *ND* not described, *r* resistant to one or more insecticides under investigation, *s* susceptible to one or more insecticides under investigation, *UC* universal coverage, *WL* wall lining

^a^Indicates traditional, permanent rural households or villages, unless otherwise specified

^b^Full coverage defined as four inner house walls, all interior surfaces in temporary structures or all interior surfaces in a λ-shaped tent, as applicable

^c^Outcomes reported relative to untreated control, unless otherwise specified

## Commercial development of insecticide-treated housing materials

**Fig. 1:**
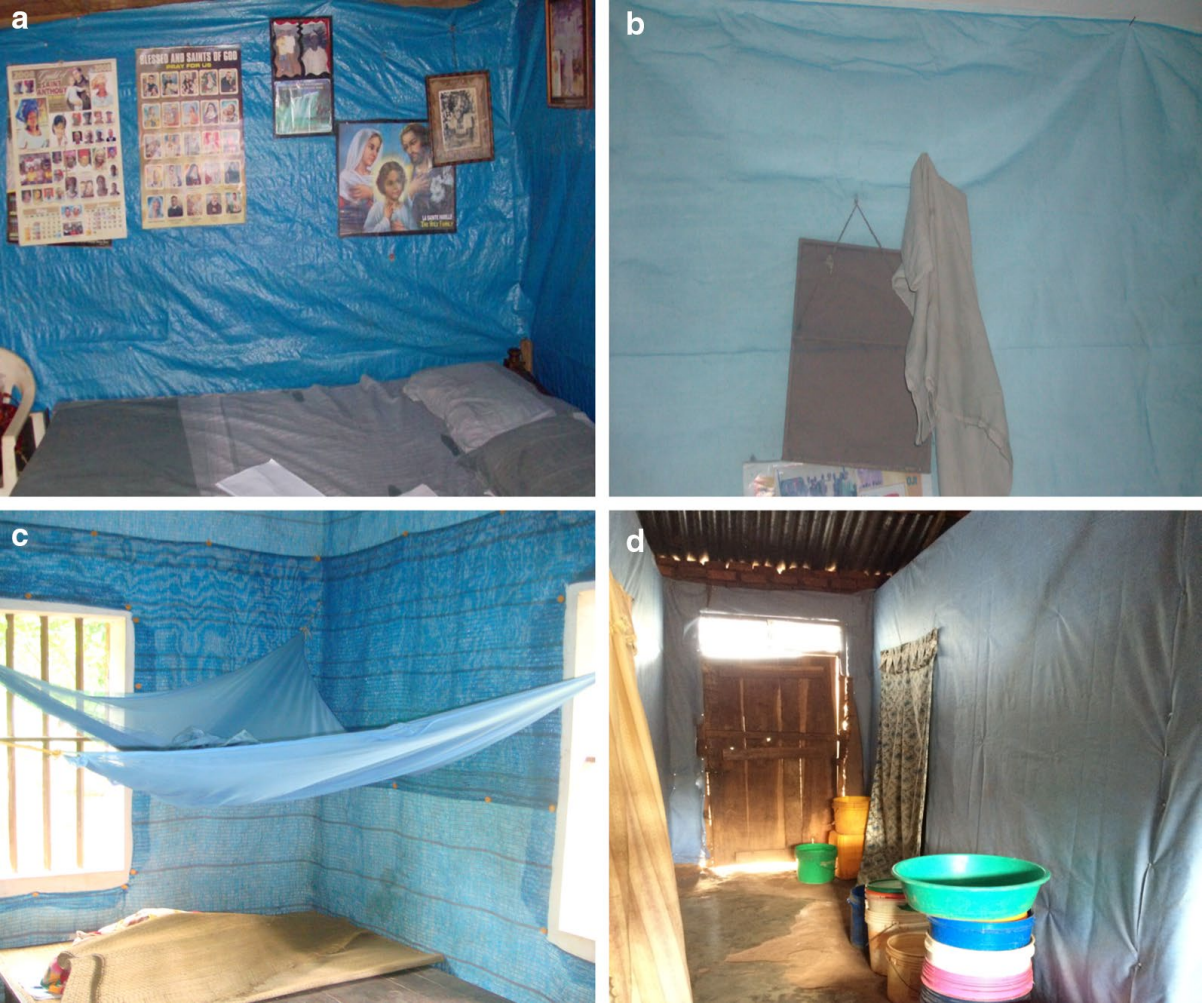
Commercial ITWL products. **a** Polyethylene plastic sheeting (ZeroFly^®^). **b** Polyester netting (PermaNet^®^ 2.0). **c** Polyethylene woven shade cloth (ZeroVector^®^). **d** Polypropylene non-woven fabric (PermaNet^®^ Lining)

The promising results demonstrated by ITPS stimulated an interest in developing a long-lasting, sustainable, community-level version for permanent use in malaria endemic settings. Such a material would offer the prospect of a novel system of insecticide delivery, which could be more residual than IRS, provide a more uniform covering of the wall with insecticide and potentially improve the interior appearance of traditional dwellings, particularly in rural areas. To identify an acceptable wall lining material, among urban and rural houses in Angola and Nigeria, three deltamethrin-treated prototypes (polyethylene woven shade cloth, laminated polyethylene plastic sheeting (ZeroFly^®^) and polyester netting (PermaNet^®^ 2.0) were assessed for their levels of household acceptability, installation feasibility and willingness to pay (Fig. 1) [52]. Rural participants highly favoured the concept of a wall lining for malaria control because of its observable impact on mosquitoes and other nuisance insects and perceived decorative value, given an existing predilection for house decorations. Of the prototype materials, polyethylene shade cloth was the most popular because of its ease of installation and resemblance to local materials. Based on these pilot field trials, the original iteration of insecticide-treated durable wall lining (henceforth ITWL; referred in previous publications as ‘durable lining’ or ‘DL’) was produced in the form of high density polyethylene woven sheets containing deltamethrin (ZeroVector^®^; 175 mg/m^2^) (Fig. 1). Initial small-scale studies across multiple African and Asian countries demonstrated consistently high levels of user acceptability, entomological efficacy and no significant loss of insecticidal activity over 1 year of household use [53, 54]. However, no phase III evaluation of this product was ever conducted due to the emergence of widespread pyrethroid resistance among vector populations across sub-Saharan Africa [6, 7]. In response, the latest generation of commercial ITWL (PermaNet^®^ Lining; Vestergaard Frandsen) was designed as a non-woven, high density polypropylene fabric containing a proprietary mixture of two non-pyrethroid insecticides (abamectin 0.25% and fenpyroximate 1%), to potentially mitigate insecticide resistance (Fig. 1). This product is currently the subject of an ongoing cluster-randomized controlled trial in an area of pyrethroid-resistance in rural North-East Tanzania, in comparison with UC of LLINs, assessing whether this version of ITWL can provide additional protection from malaria [55].

## A potential role for insecticide-treated housing materials in resistance management

Now that pyrethroid resistance is pervasive across Africa, there has been a policy shift away from pyrethroid IRS towards the restriction of this insecticide class to LLINs for which there are currently no approved alternatives [49]. Because the ‘mode of action’ of ITWL is analogous to a long-lasting IRS and Africa has become a LLIN using continent, the combined use of ITWL and LLINs may have resistance management potential. In areas with pyrethroid-resistant vector populations, the role of ITPS/ITWL plus LLINs or IRS to mitigate selection of resistant genotypes was investigated in experimental settings. Theoretically, combining interventions with different active ingredients can improve vector control because mosquitoes which are resistant to the insecticide in one intervention may be susceptible to the chemical class contained in the other. Several studies demonstrated that the combination of ITPS and LLINs can increase mortality, blood feeding inhibition and personal protection, the latter largely provided by LLINs, [41, 48], but that ITPS, when used alone, may select for resistant vectors, as evidenced by higher proportions of mosquitoes carrying resistance genes surviving in ITPS-treated huts [41, 42, 47, 48]. The difference in selection pressures likely reflects the different stages of the gonotrophic cycle, which ITPS and LLINs disrupt. Host-seeking mosquitoes upon encountering a LLIN may persist in their attempt to feed, by either making more flights between treated walls and the netted sleeper, increasing the chances of exposure to a lethal dose of the non-pyrethroid insecticide in the ITPS, or from the pyrethroid LLIN by probing for longer on the net surface, particularly if they have a degree of pyrethroid resistance and are less irritated. In this scenario, a proportion of females resistant to either insecticide would be killed. However, in the absence of a LLIN, once successfully fed, females become relatively quiescent and alight on the walls where differential selection, between susceptible and resistant genotypes, to the ITPS insecticide occurs. This explanation is plausible in Burkina Faso where resistance to the ITPS insecticide was rare and was selected by the ITPS when applied alone but not when ITPS was combined with LLINs [48]. However, in Côte d’Ivoire, where the baseline frequency of resistance to the organophosphate-containing ITPS was higher and where multiple resistance mechanisms to this chemical class were present [56], the same combination of interventions, as applied in Burkina Faso, did not significantly increase mosquito mortality rate over ITPS or LLIN alone, and did not limit the selection of resistant genotypes [47]. Hence the resistance management potential of combining ITWL and LLIN is not a foregone conclusion but appears to depend on the mechanisms and frequency of resistance already present in a locality or country as a result of previous selective pressures. These studies caution the application of ITWL in areas with resistant vectors in the absence of high community-level net coverage to safeguard continuing personal protection afforded by LLINs.

## Key determinants of community-level ITWL acceptability

The principal rationales of ITWL, which render it an attractive alternative to IRS, are its longevity, provision of protection to LLIN non-compliers and potential to overcome the user and donor fatigue associated with repeated rounds of spraying. Consequently, the majority of latterly ITWL studies have focused on identifying key determinants of acceptability and operational feasibility of implementing this intervention in endemic areas (Table 3). In general, themes of decorative value, ownership prestige, few noticeable adverse events and immediate and sustained entomological efficacy have all been reported to positively affect participant receptivity and compliance [52, 53, 57]. The relative influence of these factors on levels of community acceptability varies between study sites. In Angola, despite householders initially commending ITWL for improving their house aesthetics, once the material was considered ineffectual, the majority of participants removed theirs [52]. By contrast, in a multi-centre trial, respondents unanimously reported wanting to keep their ITWL even if it had no impact at all on mosquito populations or other nuisance insects [53]. Other attractive features of ITWL described in these studies include, the concept of a single intervention that would alleviate the daily inconvenience of multiple control measures, its role as an additional building material to block holes in walls, reduce draughts, noise and dust, and how easily it can be removed and re-installed when certain communities participate in annual house renovations, particularly re-smearing walls with mud during festive periods [57, 59]. Common aspects of ITWL which were causes for concern amongst householders were its impact on house ventilation, possible flammability, fragility, especially in the context of damage caused by children, and how long-term exposure to smoke from internal, unventilated fires may affect its aesthetics, durability and insecticidal efficacy. Finally, one more unexpected, negative outcome reported in several sites was the collateral cessation of LLIN use and other methods of disease control, as ITWL was perceived to be either a sufficient or superior malaria prevention strategy [57–59]. These observations clearly demonstrate that application of this intervention must be accompanied by re-iterative community sensitization to sustain the use of all available control measures.

**Table 3:** Summary of key determinants of insecticide-treated wall lining acceptability, identified through qualitative community surveys

Field site(s), country (sample size)	Intervention (insecticide)	Study duration	Key determinants of intervention acceptability	Supporting quotations	Additional observations	References
Huambo province, Angola (60); Enugu (60), Kano (57) and Lagos (61), Nigeria	Blue polyethylene woven shade cloth (ZeroVector^®^) (Deltamethrin)	12 months	Immediate entomological efficacy	*‘The thing is picking insects the way I can’t explain. It’s picking them like a magnet. It was very very effective.’* (Female 18–29 years, rural Enugu)	ITWL was commended for being a single preventative measure which could alleviate the use of multiple strategies incorporated into daily routine	[52]
			Aesthetic value	*‘Since we have put that thing, it has beautified my house.’* (Female 18–29 years, rural Enugu)	Higher levels of acceptability in Nigeria may be attributable to overall greater awareness of malaria and preventative measures	
	Blue laminated polyethylene plastic sheeting (ZeroFly^®^) (Deltamethrin)				Angolan participants, despite reporting positive feedback, ultimately removed their ITPS once it was perceived as ineffectual	
					A dichotomy emerged between rural and urban householders; the latter rejected the use of wall linings based on objections to their aesthetics and installation feasibility	
	Blue polyester netting (Deltamethrin)				Of the three prototype materials, ZeroVector^®^ was the most popular because of its ease of installation and resemblance to local materials	
Río Muni, Equatorial Guinea (40), Obuasi municipality, Ghana (60), Koulikoro, Mali (24), Mpumalanga South Africa (12) and Hoa Binh province, Vietnam (12)	Blue polyethylene woven shade cloth (ZeroVector^®^) (Deltamethrin)	12–15 months	Immediate and sustained entomological efficacy	*‘This fabric was very helpful because the mosquitoes have fled. The flies also leave us alone.’* (Mali, female)	Majority of participants expressed interest in keeping the ITWL for decoration even if it did not kill mosquitoes or other nuisance insects	[53]
			Aesthetic value	*‘The textile is very good because in addition to its insecticidal activity, it makes the room more beautiful.’* (Mali, female)	When offered the choice of other vector control interventions (IRS or insecticide-treated curtains), ITWL was the most popular, irrespective of earlier household allocation	
			Potential protection from malaria	*‘Since the textile arrived I have not seen a case of malaria.’* (Mali, female)		
Highland and lowland, Papua New Guinea (40)	Blue polyethylene woven shade cloth (ZeroVector^®^) (Deltamethrin)	1 month	Immediate and sustained entomological efficacy	*‘The first day after [ITPS]* ^a^ *installation I saw mosquitoes flying into the house, contact the material and then just fall off and die. The cockroaches climbed up the [ITPS covered] wall and died instantly.’* (Lowlands village, male)	Participants appreciated the ITPS acting as additional building material, blocking holes in walls, reducing draughts, noise and dust entering the house	[57]
			Potential protection from malaria	*‘We do not want to be sick with malaria. If the kids are sick, we will struggle to walk a long way to go to the hospital. We do not want this to happen. This plastic sheeting will help protect us and our children from getting sick with malaria.’* (Lowlands village, male)	Many recipients ceased LLIN use, perceiving the ITPS to be sufficient and/or superior for protection	
			Aesthetic value	*‘When I opened the door and went into the house it looked a lot different [following ITPS installation]. My house looked beautiful and was glowing.’* (Highlands urban, male)	It was difficult to establish ‘routine’ installation due to heterogeneous house size, shape and construction	
			Ownership prestige	*‘One of my sisters came and saw the durable lining sheets and liked it and said she wished she could have got one like this too.’* (Islands village, female)	Householders raised concerns about the products flammability, fragility and possibility of theft by the installation team	
			Few observable side effects	*‘I have a small child and I was worried that the insecticide on the durable lining sheet might have a bad effect on my child.’* (Islands village, female)	ITPS was exposed to smoke from internal, unventilated fires which may result in more rapid degradation, reduction in aesthetic appeal and impact insecticidal longevity and potency	
Highland and lowland, Papua New Guinea (38)	Blue polyethylene woven shade cloth (ZeroVector^®^)	36 months	Immediate and sustained aesthetic value	*‘Yes, initially it [the DL] looked very nice. It made the house look nice, but now that it is losing its colours or maybe the dust covered it so its colours are fading. But it’s still looking nice on the wall as it is.’* (Highlands urban, 36 months)	Despite reductions in perceived effectiveness over time, householders did not remove the material and most expressed interest in obtaining a new one	
					Despite reductions in perceived aesthetic value over time, householders still felt their home interior was enhanced. However, no participants expressed interest in installing a DL for appearance sake alone, suggesting perceived entomological effectiveness was important for initial and continued acceptability	
	(Deltamethrin)		Potential protection from malaria	*‘For myself, when this thing [DL] was there I see that me or my family members had never been sick with malaria since this thing was installed. Not one of us was infected with malaria. This is why I like that thing.’* (Islands village, 36 months)	Householders from the cooler highland region suggested that the material warmed the house, which was considered a desirable function. This ‘warming’ benefit was not reported by those in the lowlands	[58]
			Ease of use and perceived effectiveness compared to other malaria control methods	*‘Previously we used to do the work of tying up nets and sleep and even in the night to wake up and tie up nets and now this green net is here, sorry blue net [DL], that we do not have the hard work of tying the nets. It’s [DL] on the wall helping us to kill mosquitoes so we just sleep relaxing’* (Highlands village, 36 months)	Many recipients reported ceasing LLIN use, perceiving the ITPS to be sufficient and/or superior for protection. Householders made no indication to suggest awareness that this reduction in net use might increase risk of malaria	
				*‘[DL] is better than the mosquito net and the other thing is that I can breathe properly when I’m sleeping, but in the mosquito net I feel that I am breathing in all the medicine/treatment from the net. Now that we are using this [the DL], we don’t want to use the mosquito net, our nets are piling up there. I am ready to sell mine. We don’t really like mosquito nets. These nets [DL] are better than mosquito nets. For me and my families good I’m saying this.’* (Islands village, 12 months)		
			Few observable side effects compared to other malaria control methods	*‘I don’t like using the mosquito net. Sometimes I have shortness of breath.’* (Islands village, 12 months)	Due to the type of housing material used in PNG, DL durability may be longer than average house lifespan, suggesting that effective duration would be largely determined by the age and condition of the house at the time of installation, rather than the product itself	
					Bioefficacy testing demonstrated no loss in insecticidal activity after 36 months indicating that participant perceptions of reduced product effectiveness are not necessarily synonymous with actual ineffectiveness	
Limpopo province, South Africa (40)	Green, orange, brown, or purple polyethylene monofilament (deltamethrin or alpha-cypermethrin)	6 months	Immediate and sustained entomological efficacy	*‘Net* ^*b*^ *helps a lot because there are not as many mosquitoes like before. Like nets for both rooms.’* (Female, age 28, house with green lining)	Majority of participants ceased using other methods to prevent malaria, including spraying insecticides and burning mosquito coils and other materials	[59]
				‘The *net is helping us because mosquitoes are not as many as before when there is no lining.’* (Female, age 73, hut with brown lining)	Householders disagreed over whether ITWL should cover the entire wall or only the top portion, out of reach of children and potential damage	
					Smoke damage and soot accumulation from cooking over open, unventilated fires was raised as an issue which might impact ITWL long-term aesthetic appeal and insecticidal efficacy	
			Aesthetic value	‘The *net is too much good. It decorate my room and it kill mosquitoes and cockroaches.’* (Female, age 48, house with orange lining)	The ability to remove and re-install ITWL would overcome logistical problems associated with IRS in the area, namely the annual or bi-annual mud re-smearing, re-painting or washing of walls that occurs during the festive season	

*DL* durable lining, *IRS* indoor residual spraying, *ITPS* Insecticide-treated plastic sheeting, *ITWL* insecticide-treated durable wall lining, *LLIN* long-lasting insecticidal net

^a^In the study by Pulford et al. ITPS is used to refer to ZeroVector^®^ ITWL, not to ZeroFly^®^ ITPS

^b^In the study by Kruger et al. net is used to refer to the mesh ITWL, not to LLINs or other mosquito nets

## Future prospects of ITWL for malaria control: control intervention or method of house improvement?

In the absence of unequivocal evidence to support ITWL as an alternate control measure to IRS, the questions remain, how will this intervention function to reduce malaria, in what epidemiological situation will it warrant implementation and how will it be executed to scale? There is increasing evidence to support a crucial role for housing improvement in malaria control [31–33, 60, 61]. It can be envisaged that ITWL could act as an effective and insecticidal method of house, and in particular, eave screening, if affixed to the base of the roof or ceiling and proven to have long-term durability. However, with concomitant housing, social and economic development, will potential communities still accept ITWL as readily based on its perceived aesthetics? Reports from more affluent urban residents in Nigeria suggest this might not be the case [50]. Alternatively, even if ITWL were to be proven effective and applied in a similar manner to IRS, there are considerable implications for installation logistics. Previously, ITWL has been primarily installed using locally-sourced nails, often covered with plastic caps to improve wall grip [62]. Installation time, which accounts for time taken to attach the material to house walls, as well as preparation (removal of all household and wall items) and clean-up, is largely correlated with overall house size, construction and number of rooms to be covered. From an economic perspective, lengthy or highly variable installation times, among communities containing heterogeneous house constructions, will have repercussions on intervention cost-effectiveness, potentially requiring financing mechanisms that many African countries lack [63]. By comparison to IRS, which is estimated at as little as $5 for pyrethroid (ICON™ lambdacyhalothrin capsule suspensions) to $23.50 for organophosphate sachets (Actellic CS 3000) [64], ITWL installation also requires the purchase, temporary storage and transportation of large ITWL rolls (measuring 2.4 × 210 m and weighing 40 kg each), supporting fixings and resources (e.g. nails, hammers, tape measures, step ladders etc.), often to remote and inaccessible locations. In this scenario, unlike IRS, the cost of contracting and deploying specialist installation teams by NMCPs would likely be financially prohibitive.

Other, as yet unanswered issues, include just how much of a wall or house must be covered with ITWL to impact disease transmission, could ITWL coverage be restricted to sleeping rooms with only limited loss of effectiveness and how can high quality intervention installation and community maintenance be ensured and monitored, as ITWL is expected to function for multiple years, without external upkeep or interference. Moreover, should ITWL durability be assessed in terms of overall household-level coverage, given it will likely impact malaria transmission like IRS, through a reduction in overall vector population density, or because of its long-lasting LLIN-like properties, will the formation of holes from daily household wear and tear also impact efficacy? Given its higher cost, ITWL is unlikely to be considered for widespread programmatic implementation but instead may be more appropriate as a method to control malaria in areas where pyrethroid-resistant vectors predominate, or to reduce epidemic hot spots of transmission [20, 65]. Unlike vertical IRS programmes and mass LLIN distributions, potential delivery systems for ITWL could utilize a combination of social mobilization and microfinancing or subsidization, designating direct responsibility of installation and maintenance to community members.

## Future prospects of ITWL for control of other vector-borne diseases

To date, ITWL has primarily been evaluated for its effectiveness as a malaria control strategy. However, there are fundamental features underlying the biology of other vector-borne diseases where ITWL could also play a critical role in interrupting disease transmission. Leishmaniasis remains an important neglected tropical disease with an estimated 350 million individuals at risk worldwide [66]. Vector management is one of the principal disease control strategies, targeting putative resting sites of phlebotomine sand flies, usually with IRS [67]. In addition to all of the aforementioned limitations of IRS, because some vector species display crepuscular feeding activities, LLINs can also be ineffective in these endemic countries [68]. Recently, the efficacy of ZeroVector^®^ ITWL was investigated in a multi-centre study in Bangladesh, India and Nepal, demonstrating high levels of sand fly mortality and household acceptability and decreases in vector density over 12 months of household use [69, 70]. However, no epidemiological endpoints to assess the impact of ITWL on incidences of visceral leishmaniasis were measured, indicating further evaluations of this intervention are still needed. ITWL also warrants consideration as a supplementary intervention to control Chagas disease, which is transmitted by highly domiciliated triatomine bug vectors, inhabiting cracks in the walls of rural adobe houses across Latin America [71]. Despite achieving substantial reductions in disease incidence through historic large-scale trans-national IRS campaigns, active transmission persists, particularly in the Gran Chaco, where rapid domestic re-infestation abounds and insecticide resistance is increasing; both of which are exacerbated by decentralized regional control efforts in areas of recurrent political, social and economic instability [72]. While ITWL has yet to be directly evaluated against Chagas disease, organophosphate and juvenile growth hormone containing insecticidal vinyl paints (Inesfly 5A IGR^®^), based on similar principles to ITWL, have thus far reported encouraging experimental results [73, 74] and long-term reductions in levels of household triatomine infestation [75, 76].

## Conclusions

Insecticide-treated durable wall lining (ITWL) is a novel method of vector control, which when attached to inner house walls remains efficacious over multiple years and can circumvent some of the logistical constraints associated with first-line control strategies. To date, there is substantial phase II data indicating ITWL can impact malaria vector populations, with complete wall coverage affording the highest rates of mosquito mortality, deterrence and blood-feeding inhibition in experimental hut trials. However, there is currently limited Phase III evidence to support operational implementation of ITWL either as a control intervention in a programmatic context or as an insecticidal method of house improvement or eave screening. While aesthetic value and observable entomological efficacy are key determinants of acceptability, additional studies are still required to determine feasible and cost-effective financing mechanisms of installation to sustain ITWL durability during long-term field use. Further large-scale community-level trials are warranted to support the development and evaluation of ITWL as a potential alternate control strategy for malaria and other vector-borne diseases.

## Abbreviations

CRT: cluster-randomized controlled trial
CS: capsule suspension
DL: durable lining
IGR: insect growth regulator
ITN: insecticide-treated net
IRS: indoor residual spraying
ITPS: insecticide-treated plastic sheeting
ITWL: insecticide-treated durable wall lining
LLIN: long-lasting insecticidal net
NMCP: National Malaria Control Programme
UC: universal coverage
